# Special Issue: New Research on Bioactive Natural Products

**DOI:** 10.3390/ijms26199408

**Published:** 2025-09-26

**Authors:** Alexandros Tsoupras

**Affiliations:** Hephaestus Laboratory, School of Chemistry, Faculty of Sciences, Democritus University of Thrace, Kavala University Campus, 65404 Kavala, Greece; atsoupras@chem.duth.gr

Chronic disorders, including cardiovascular diseases [1], cancer [1,2], renal and neurodegenerative conditions [1,3], allergies [4], autoimmune diseases [5], and persistent infections like HIV infection [6], periodontitis [7], sepsis [8], and SARS-CoV-2 infection [9], remain among the most significant global health burdens and challenges. Although current preventive and therapeutic approaches are often effective, they are frequently associated with high financial costs, adverse side effects, and sustainability concerns. This reality has intensified the demand for innovative strategies centered on natural bioactives derived from sustainable sources, which offer a promising alternative for the development of natural products with health-promoting properties against these disorders, including functional foods, nutraceuticals, cosmeceuticals, and novel drug candidates [1,2,3,5,9,10,11,12,13,14,15,16,17,18,19,20].

This Special Issue, “New Research on Bioactive Natural Products”, highlights recent advances in the discovery, characterization, and application of natural compounds with health-promoting properties. More specifically, the aim of this Special Issue was to advance research in this field by exploring the valorization of natural bioactives from diverse sources such as microbial bio-cultures and agro-food by-products. Emphasis was placed on state-of-the-art isolation, structural characterization, and bioassays, as well as translational studies assessing efficacy, safety, and bioavailability.

The contributions of articles include cutting edge research and reviews on computational systems biology approaches for herbal medicines, innovative 3D-printed dosage forms, plant cell culture-derived cosmeceuticals, chemically modified flavonoid derivatives with antimicrobial activity, and valorization of agri-food by-products such as apple pomace.

The articles assembled in this collection highlight the promising role of natural bioactives as core ingredients for functional supplements, nutraceuticals, cosmeceuticals, and even drug discovery, with particular relevance to inflammation-related chronic diseases. Collectively, these studies demonstrate the importance of multi-target strategies, sustainable bioproduction, and advanced delivery technologies in unlocking the therapeutic potential of natural bioactives. This Special Issue links biochemical mechanistic molecular and cellular circuits of the modes of action of natural bioactives, while also emphasizing how interdisciplinary innovation can transform them into next-generation health-promoting solutions. Future directions include integrating omics-based characterization with translational research and modern toxicological evaluations to ensure efficacy, safety, and industrial scalability.

More Specifically, Lee et al. introduced a multiscale network analysis approach to identify herbal remedies for major depressive disorder (MDD) [21]. By applying biased random walk methodologies to herb–target–disease networks, they revealed herbs such as Ephedrae herba and Glehniae radix as promising candidates. The study emphasized the therapeutic importance of multi-target synergism in complex disorders like MDD, highlighting active compounds including ephedrine, psoralen, and ursolic acid. This system-level approach exemplifies how computational methods can accelerate the discovery of herbal interventions tailored to multifactorial diseases. All in all, this study advances the existing body of research in these fields [22].

Koshovyi et al. investigated the phytochemical composition and pharmacological activity of *Matricaria chamomilla* (German chamomile) flower extracts, with a focus on novel technological applications [23]. In addition to identifying 22 polyphenolic compounds and 14 amino acids, their study demonstrated analgesic and soporific effects potentiated by amino acid co-formulations such as glycine and lysine. Molecular docking suggested modulation of GABAA_A and NMDA receptors. Notably, the authors developed polyethylene oxide-based printing gels for 3D-printed oral dosage forms, underscoring the potential of advanced manufacturing in nutraceutical delivery systems. Overall, this work extends prior investigations conducted in these areas [24].

Kim et al. analyzed *Freesia refracta* callus extract (FCE), characterizing its bioactive composition using advanced chromatography and spectroscopy methods [25]. The study identified compounds such as nicotinamide and pyroglutamic acid, which demonstrated strong antioxidant properties and efficacy in promoting collagen synthesis. In vitro and in vivo results showed significant improvements in fibroblast–collagen interactions and skin smoothness, with measurable anti-aging benefits in human volunteers. This work supports the cosmeceutical potential of plant cell culture-derived extracts in enhancing skin health. Taking the above into account, this work enhances and expands the current state of research in these disciplines [26].

Perz et al. explored chemically synthesized and biotransformed flavonoid derivatives containing chlorine, bromine, and nitro groups [27]. The derivatives displayed distinct antimicrobial activities, with chlorinated and nitrated flavones exhibiting potent inhibition of pathogenic bacteria. Interestingly, certain flavanones stimulated probiotic bacterial growth, revealing potential applications in microbiome-targeted therapies. Their results highlight how structural modifications and fungal biotransformations can enhance the pharmacological spectrum of flavonoids. Based on the above, this study contributes to the progression of knowledge within these fields [28]

Vandorou et al. provided a comprehensive review on the valorization of apple pomace, a by-product of apple processing [29]. Apple pomace extracts, which are rich in natural bioactives, including polar lipids rich in unsaturated fatty acids, phenolic bioactives, vitamins, minerals, and dietary fibers, demonstrated antioxidant, anti-inflammatory, anti-thrombotic, and anti-aging properties. The review summarized their mechanisms of action, synergistic effects, and practical applications in both functional foods and cosmetics. Beyond therapeutic potential, this research underscores the importance of sustainable circular economy practices in transforming agri-food waste into high-value bioactive resources. Overall, this study is an important paradigm of a holistic overview of natural bioactives from agri-food by-products and further extends prior investigations into the applicability of such bioactives as health-promoting products against inflammation-related disorders [1,2,3,5,9,10,11,12,13,14,15,16,17,18,19,20].

Collectively, the contributions to this Special Issue highlight a multifaceted trajectory for natural bioactives research. Several trends that emerge are as follows:

1. Systems Biology and Network Pharmacology—computational and network-based approaches enable a deeper understanding of the multi-target effects of natural compounds.
2. Advanced Manufacturing and Delivery—techniques such as 3D printing enhance the precision and personalization of nutraceutical formulations.
3. Plant Cell Cultures and Sustainable Sources—innovative cultivation methods expand the range of accessible bioactives while reducing reliance on endangered or resource-intensive sources.
4. Chemical Modifications and Biotransformation—structural tailoring of natural products can improve efficacy, specificity, and microbiome compatibility.
5. Valorization of Agri-Food By-Products—integrating bioactives from waste streams supports sustainability while providing cost-effective bioactive raw materials for innovative functional products in a circular economy design.

The convergence of these strategies not only addresses health-related challenges but also aligns with consumer demand for sustainable, safe, and effective natural products.

Overall, this Special Issue illustrates the breadth and depth of ongoing research into bioactive natural products, spanning computational drug discovery, phytochemistry, pharmacology, cosmeceuticals, and sustainable valorization of bioactives from agri-food bio-wastes. Together, the studies demonstrate how cutting-edge science and technology can unlock the potential of natural bioactives to prevent and manage chronic disorders, while advancing sustainability and innovation in the healthcare and nutraceutical sectors.
